# Photoacoustic tomography versus cone-beam computed tomography versus micro-computed tomography: Accuracy of 3D reconstructions of human teeth

**DOI:** 10.1371/journal.pone.0274818

**Published:** 2022-12-19

**Authors:** Sonja Jasmin Maria Schneider, Christian Höhne, Martin Schneider, Marc Schmitter

**Affiliations:** 1 Department of Prosthetics, Wuerzburg University Hospital, Wuerzburg, Bavaria, Germany; 2 Department of Bioengineering, Stanford University, School of Medicine, Stanford, California, United States of America; Polytechnic University of Marche, ITALY

## Abstract

**Objectives:**

In this in-vitro study, teeth were imaged using photoacoustic tomography (PAT), cone-beam computed tomography (CBCT), and micro-computed tomography (μ-CT). The study had aim: to identify the best wavelength for PAT images to determine the accuracy of the three imaging methods, and to determine whether PAT images of teeth can achieve acceptable reconstruction quality.

**Methods:**

Nineteen human mandibular single-rooted incisors were extracted from patients with trauma or periodontitis. To determine the best wavelength for acquiring photoacoustic images, all 19 teeth were scanned in vitro with PAT, using different laser wavelengths between 680 and 960 nm. The images were analyzed using image analysis software. To assess the accuracy of PAT and compare it with the accuracy of CBCT, each tooth was also scanned in vitro using CBCT and the reference standard technique of μ-CT. Subsequently, three different three-dimensional models, one for each imaging technique, were created for each tooth. Finally, the three different three-dimensional models acquired for the same tooth were matched and analyzed regarding volume and surface.

**Results:**

The highest quality tooth images were achieved using the 680 nm wavelength, which showed the best contrast ratio. The full geometry of the dental root (μ-CT compared with PAT) could be visualized with relative standard deviations of 0.12 mm for the surface and −7.33 mm^3^ for the volume (n = 19). The full geometry of the dental root (μ-CT compared with CBCT) could be visualized with relative standard deviations of 0.06 mm for the surface and −14.56 mm^3^ for the volume (n = 19). The difference between the PAT–μ-CT group and CBCT–μ-CT group regarding the total average of the root surface area was not significant (p>0.06).

**Conclusion:**

Images, which were acquired using PAT at 680nm showed the best contrast ration, enabling the identification of dentin, cementum and the dental pulp. No significant differences were found between the PAT–μ-CT group and CBCT–μ-CT group regarding the total average of the RSA and the total volume. Thus, three-dimensional reconstructions based on in-vitro PAT are already of acceptable reconstruction quality.

## 1 Introduction

Conventional imaging modalities in general dental practice include numerous radiographic techniques for diagnosing several pathologies of the teeth and maxillofacial bones [1]. However, conventional dental imaging techniques (intraoral radiographs or panoramic imaging [2]) have two major disadvantages: (1) interposition of anatomical parts and (2) poor detail on conventional radiography (due to under/overexposure [3]). To overcome these limitations, in the last two decades dentists have increasingly turned to three-dimensional reconstruction of teeth [4]. Several dental imaging devices are now available to create three-dimensional reconstructions [5]. Among them, micro-computed tomography (μ-CT) has recently been introduced to obtain, in a non-invasive and non-destructive way, qualitatively and quantitatively valuable information of three-dimension reconstructions of tooth [6]. However, the imaging techniques commonly used in dental practice to create three-dimensional images are still based on X-rays. Although these techniques can overcome the interposition of anatomical parts and poor detail on images, ionizing radiation still poses a serious problem because of its ability to cause temporary and permanent tissue damage [7–10].

Consequently, new imaging techniques that utilize non-ionizing wavelengths and can therefore be used frequently and repeatedly are highly desirable. Such imaging techniques could permit real-time imaging during dental operations and might help to significantly improve treatment outcomes. Furthermore, close follow-ups to evaluate treatment success and disease progression would enable the creation of new optimized treatment protocols.

Non-ionizing imaging techniques such as ultrasound (US) [11], magnetic resonance imaging (MRI) [12], optical imaging [13], and photoacoustics have been already used to create three-dimensional images of tissue [14]. Photoacoustic tomography (PAT) is an innovative and promising imaging technique. Unlike US, MRI, and optical imaging, it is based on sound and non-ionizing light in the near-infrared range [14]. This combination of light and sound ensures a higher scalability of spatial resolution and depth penetration, because acoustic waves are less scattered in tissue than photons are [14]. This emerging technique has been tested on various biological tissues in several diseases (e.g., breast cancer, atherosclerosis). Most of these applications exploit endogenous chromophores such as hemoglobin, melanin, and lipids, which possess specific absorption spectra [14]. Although, there are some reports about frequencies used to use photoacoustic on teeth [15], there is no systematic approach to identifying suitable laser wavelengths for imaging the hard tissue of complete and intact human teeth (without pathological conditions as e. g. caries) using PAT. Furthermore, the identification of the ideal wavelength for the device used in the present study (MSOT inVision 256) was mandatory as it was the basis to acquire high quality images, which should be compared with other imaging modalities, including a refence standard.

The objectives of this study are:

1. to determine the best wavelength for acquiring photoacoustic images of the teeth, and
2. to create three-dimensional models based on PAT and CBCT, and to compare them with models based on the reference imaging method μ-CT with respect to volume and surface deviations.

The null hypotheses are that:

1. the total volume of human mandibular incisors assessed in vitro by using PAT and CBCT does not significantly differ from the total volume assessed by using μ-CT, and
2. the surface area between μ-CT and CBCT, and between μ-CT and PAT does not differ significantly.

## 2 Material and methods

### 2.1 Sample preparation

This study was conducted in compliance with the Declaration of Helsinki, and the use of extracted human teeth was approved by the Medical Ethics Committee of the University of Wuerzburg (application number 15/15). All patients provided written informed consent for the use of their teeth.

Nineteen human single-rooted incisors were extracted from the mandible of patients with trauma or periodontitis. After extraction, the teeth were cleaned by means of an ultrasonic scaler (SONICflex LUX 2000L, KAVO; Biberach, Germany). After cleaning, the teeth were stored for seven months in a 1% chloramine-T solution at 5ºC to prevent bacterial colonization [16].

### 2.2 Experimental setup: μ-CT

**Fig 1:**
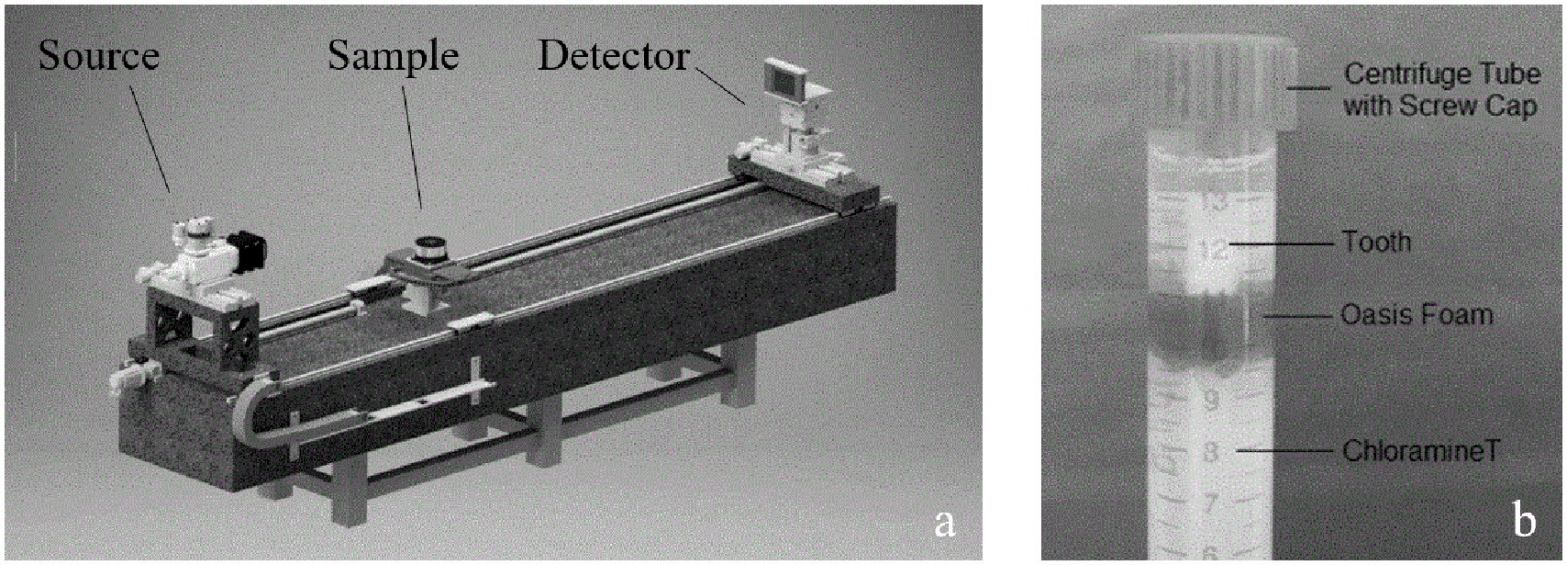
MetRIC (Micro and Region of Interest CT). To create μ-CT images, teeth were scanned with the MetRIC. **(a)** Schematic image of the MetRIC. With permission from the Institute for X-ray Microscopy, Fraunhofer IIS; Wuerzburg, Germany. **(b)** Tooth placed on sample holder to acquire μ-CT images.

To acquire μ-CT images, the teeth were scanned using a μ-CT device (MetRIC: Micro and Region of Interest CT, Fraunhofer IIS; Wuerzburg, Germany, Fig 1a). The setup for μ-CT acquisition, which compromises source, sample, and detector (anode material: tungsten), is shown in Fig 1a.

To acquire μ-CT images, each tooth was held in place with oasis foam (Gravidus GmbH; Bremen, Germany) in a screw-capped centrifuge tube that was filled with 1% chloramine-T solution (Fig 1b). For image acquisition, the image-acquisition parameters were set to 120 kV, 3 W (= 25 muA) and an exposure time of 150 ms. μ-CT acquisition resulted in a stack of two-dimensional images (voxel size: 2 μm) for each tooth, which was saved in tagged image file format (TIFF).

### 2.3 Experimental setup: CBCT

**Fig 2:**
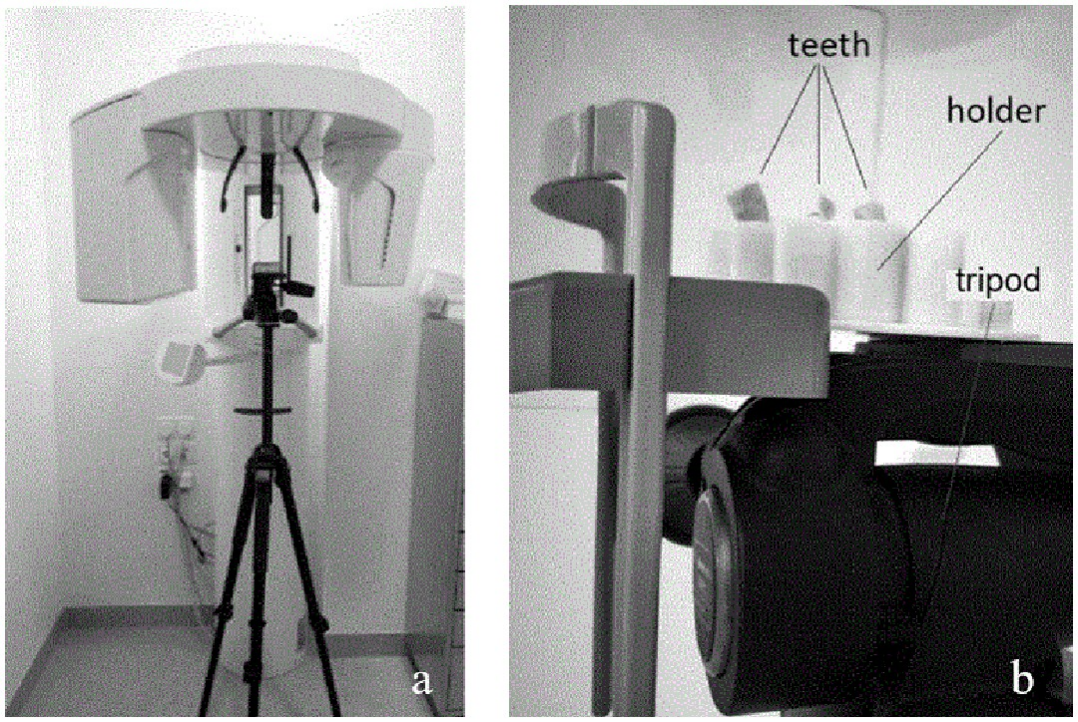
Experimental setup to create CBCT images. **(a)** Overview image of the CBCT device (Orthophos XG 3D, Dentsply Sirona; York, PA, USA) and tripod. **(b)** Detailed image of the tripod with plastic holder and teeth.

To acquire CBCT images, the Orthophos XG 3D (Dentsply Sirona; York, PA, USA, Fig 2a) was used. Before CBCT imaging, the teeth were fixed in place in a plastic holder. The holder was specifically designed for this study in the software “Cubify Design” (3D Systems; Rock Hill, USA) and printed in polyethylene terephthalate using the “German RepRap X350” three-dimensional printer (ccd systems; Berlin, Germany). The holder was then placed on a tripod to bring the teeth to the right height (Fig 2b).

For image acquisition, the image-acquisition parameters were set to high definition (voxel size 160 μm; field of view Ø 8 × 8 cm). CBCT acquisition resulted in a stack of two-dimensional images of each tooth, which were saved as TIFF files.

### 2.4 Experimental setup: PAT

**Fig 3:**
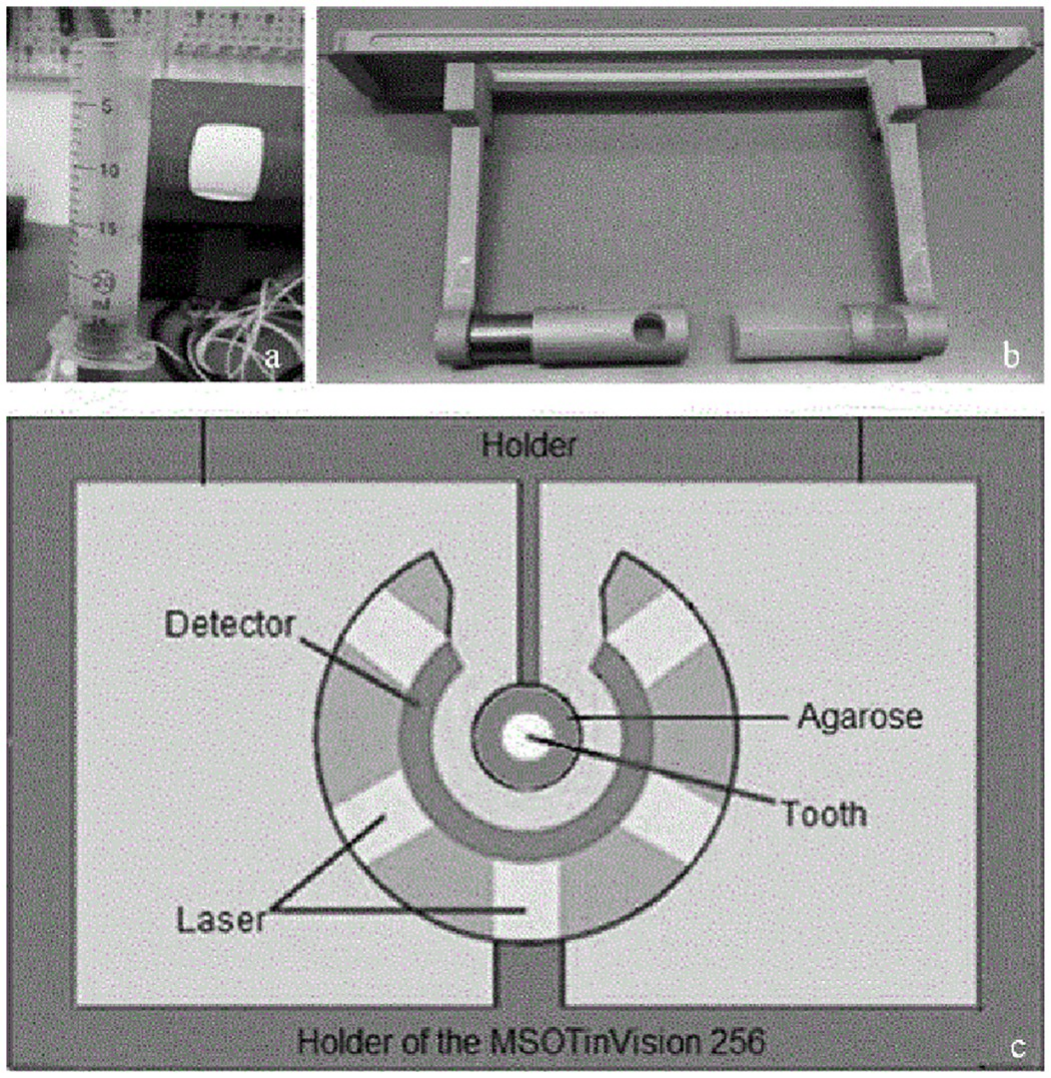
Experimental setup for PAT images. **(a)** The tooth was embedded in a solution of agarose and lipofundin in a plastic syringe. **(b)** Afterwards, the holder containing the solidified agarose and lipofundin mixture was attached to the MSOT inVision 256. **(c)** Schematic of the interior components of the MSOT inVision 256.

To create the PAT images, the MSOT inVision 256 (Multispectral Optoacoustic Tomography, iThera Medical GmbH; Munich, Germany, Fig 3) was used. Each tooth was embedded in a 20 ml plastic syringe, in a solution of 2% agarose with 1% lipofundin (Intralipid). The agarose was used to fix the tooth in a suitable position, and the lipofundin ensured better light scattering. Fig 3a shows how the tooth was fixed in the agarose and lipofundin mixture, and Fig 3b shows the MSOT inVision holder. A schematic of the components of the MSOT is shown in Fig 3c.

For PAT imaging, the MSOT inVision was filled with warm water (25ºC), and the step size (distance between one picture to the next) was set to 0.3 mm (data filter 50 kHz– 5 MHz; resolution 150 μm). An algorithm based on back-projection was used for image reconstruction. Back-projection reconstruction is a very fast and semi-quantitative reconstruction algorithm that allows efficient generation of anatomical images [17].

After being fixed in place, teeth were scanned at wavelengths of 680–960 nm, in 10 nm increments. Depending on the size of the individual tooth, 9–9.3 minutes were needed to record each tooth. PAT image acquisition resulted in a stack of two-dimensional images of each tooth, which were saved as a TIFF file.

After data acquisition, the images were analyzed to determine the imaging quality of each wavelength.

### 2.5 Data analysis

First, the most suited wavelength for PAT when imaging teeth was identified by using image analysis software (ImageJ2, public domain software): the distribution of the grey values was analyzed by choosing “Plot Profile”. The grey values and their distribution stand for the contrast of the images. The grey values were analyzed further using the Kruskal-Wallis-Test and the post-hoc Mann-Whitney U-test.

For data analysis, the volume and surface data for PAT and CBCT were compared with the corresponding data from μ-CT, with special emphasis on the dental root. We chose μ-CT images as the reference standard because the spatial resolution of μ-CT (up to 2 μm) is more accurate than that of CBCT (160 μm) and PAT (150 μm).

The root surface deviation (RSD) and root volume deviation (RVD) between the PAT and μ-CT images, and between the CBCT and μ-CT images were calculated and analyzed. To calculate the RSD, the total average of the RSD (average of the positive and negative RSD values) was determined.

The following standardized protocol was used when creating and matching the three-dimensional models:

First, the acquired two-dimensional images (TIFF files) of the incisors were assembled and compiled in three-dimensional models in version 4.10.1 of “3D Slicer,” a proprietary software program written in Matlab 2010b (MathWorks; Natick, Massachusetts, USA).

Second, GOM Inspect was used to match the PAT images with the μ-CT images.

The GOM Inspect software is used by the industry in product development, quality control and production with a focus on alignment and deviation measurement. The software is certified by NIST (National Institute of Standards and Technology, Gaithersburg, Maryland, United States) and PTB (Physikalisch-Technische Bundesanstalt, Braunschweig und Berlin). GOM Inspect has been placed in Category 1 with the smallest measurement deviations and was used in many studies [18]. GOM was used with a double stage automized matching process.

In the first stage GOM performed a preliminary matching process. In the second stage the automized precision matching was performed. Subsequently, the software program Fusion 360TM (2020 Autodesk, Inc.) was applied to crop the three-dimensional models of the same mandibular incisor to the same size. Afterwards, the volume of the root was calculated by applying the software program Meshmixer 3.5.474 (2017 Autodesk, Inc.).

Once the three-dimensional models had been matched and cropped, the total average of the deviation, the normalized total average of the deviation, the positive deviation, and the negative deviation of the root surface area (RSA) were calculated, using μ-CT as the reference standard. In addition, the total volume of the root was determined and compared among the three models.

### 2.6 Statistical evaluation

Data were analyzed in the software program SPSS Statistics (Statistical Package for the Social Sciences, version 25.0.0; Armonk, USA). Several statistical tests were used, which are described in the following subsections.

#### 2.6.1 Frequency distribution

As a first step, the normal distribution of the data was analyzed using the Kolmogorov–Smirnov test. This test compares the observed cumulative distribution of scores with the theoretical cumulative distribution for a normally distributed population [19].

#### 2.6.2 Assessment of differences between groups

Because not all groups showed a normal distribution, a non-parametric test was applied to assess the significance level. For this purpose, Kruskal-Wallis test and Mann–Whitney U test was applied [20]. In the present study, the level of significance was set at ≤ 0.05 (highly significant: p-value ≤ 0.01) [21].

## 3. Results

**Fig 4:**
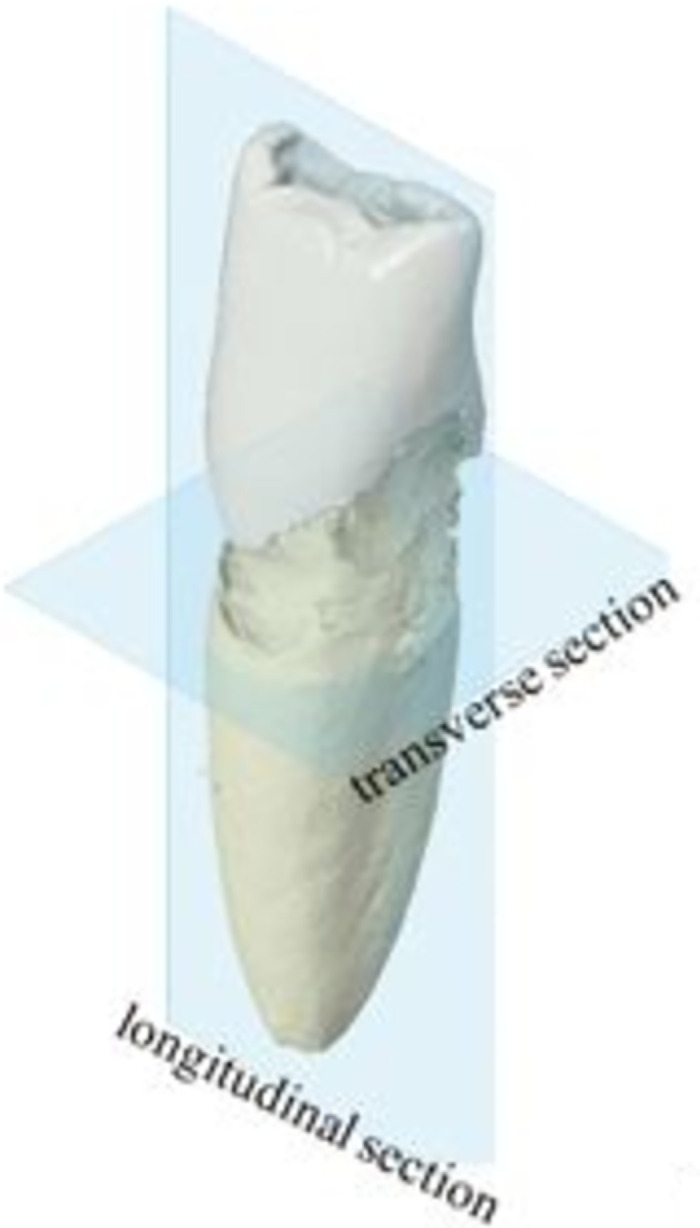
Longitudinal and transverse section relative to the whole tooth anatomy.

The following figures show longitudinal and transverse section of the tooth (Fig 4).

**Fig 5:**
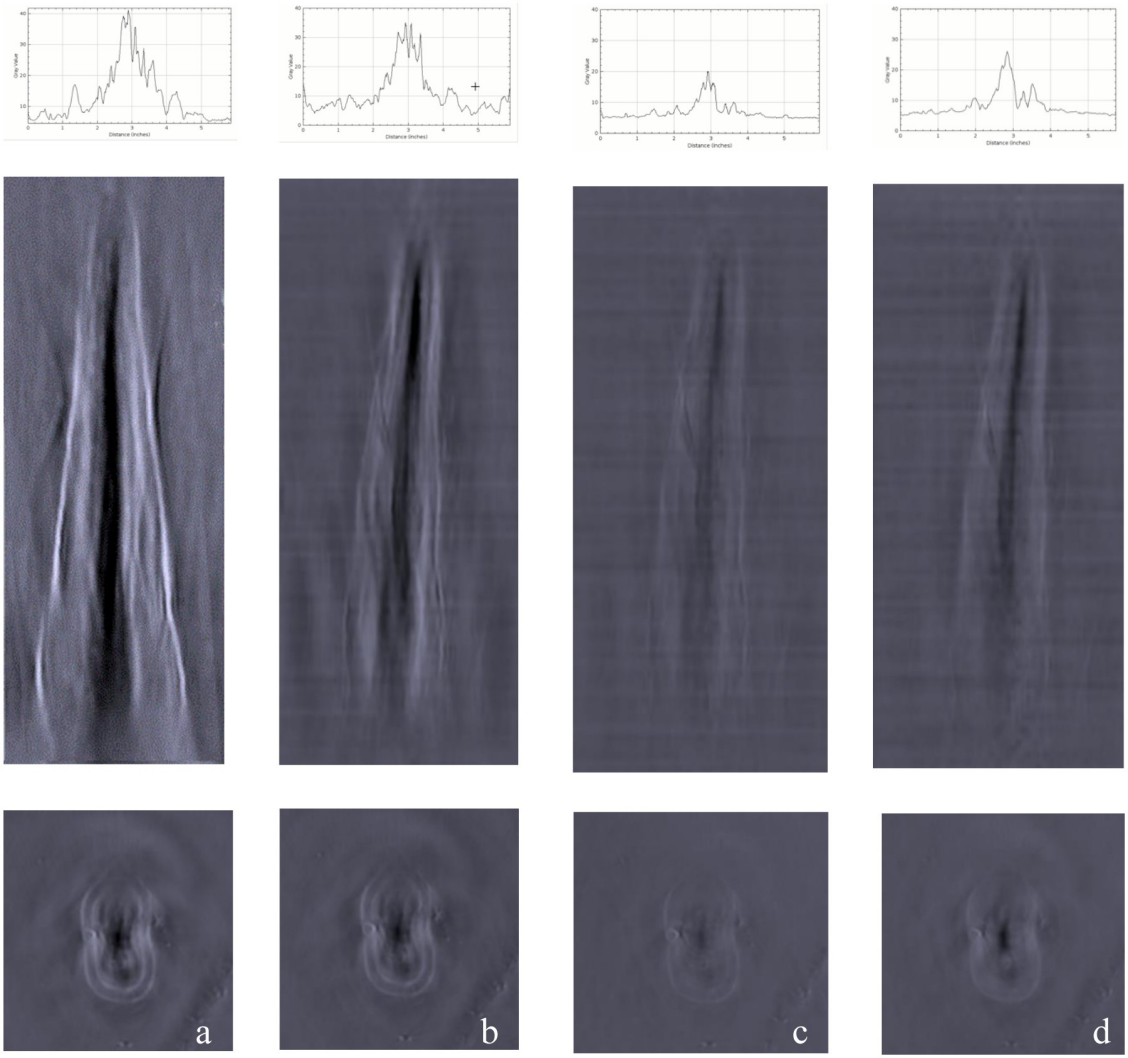
Comparison of photoacoustic images of the same human mandibular incisor recorded with different wavelengths. The analysis of the grey-scale distribution with respect to the longitudinal sections are given in the graphs. The images show the longitudinal and transverse section of the incisor. **(a)** 680 nm, **(b)** 700 nm, **(c)** 800 nm, **(d)** 900 nm.

The analysis showed that a wavelength of 680 nm produced the best contrast (assessed using grey values) when imaging extracted human mandibular incisors with PAT (Fig 5). Kruskal-Wallis test showed, that there are significant differences between the groups with respect to the contrast ratio. Post-hoc Mann-Whitney U-Test showed significant differences (p<0.05) between 680nm and 800nm/900nm and between 700nm and 800nm. At 680nm wavelength, a high contrast signal was detected that could be clearly distinguished from the background. Photoacoustic images acquired with a wavelength over 680 nm showed a higher incidence of discontinuous and incomplete lines, accompanied by a reduced contrast.

**Fig 6:**
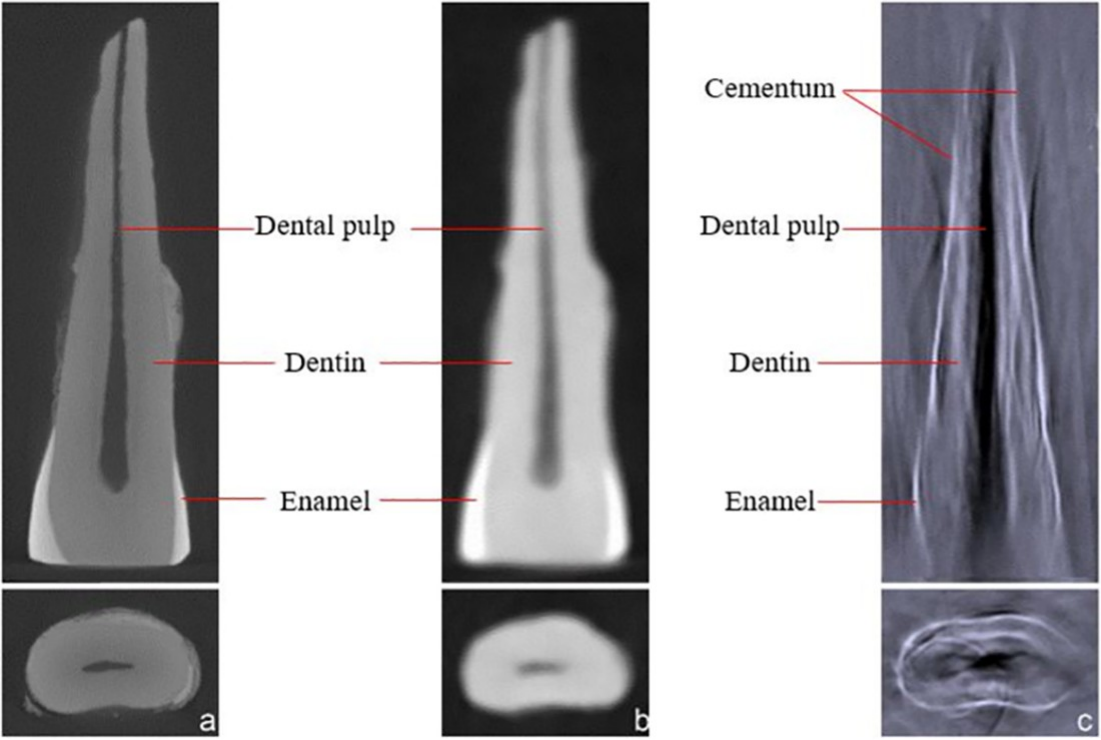
Comparison of μ-CT, CBCT, and PAT images. The images show the longitudinal and transverse section of a human mandibular incisor. **(a)** μ-CT image, **(b)** CBCT image, **(c)** PAT image (680 nm).

An example for the results of the comparison of acquired two-dimensional images (TIFF files) of the incisors is given in Fig 6.

**Fig 7:**
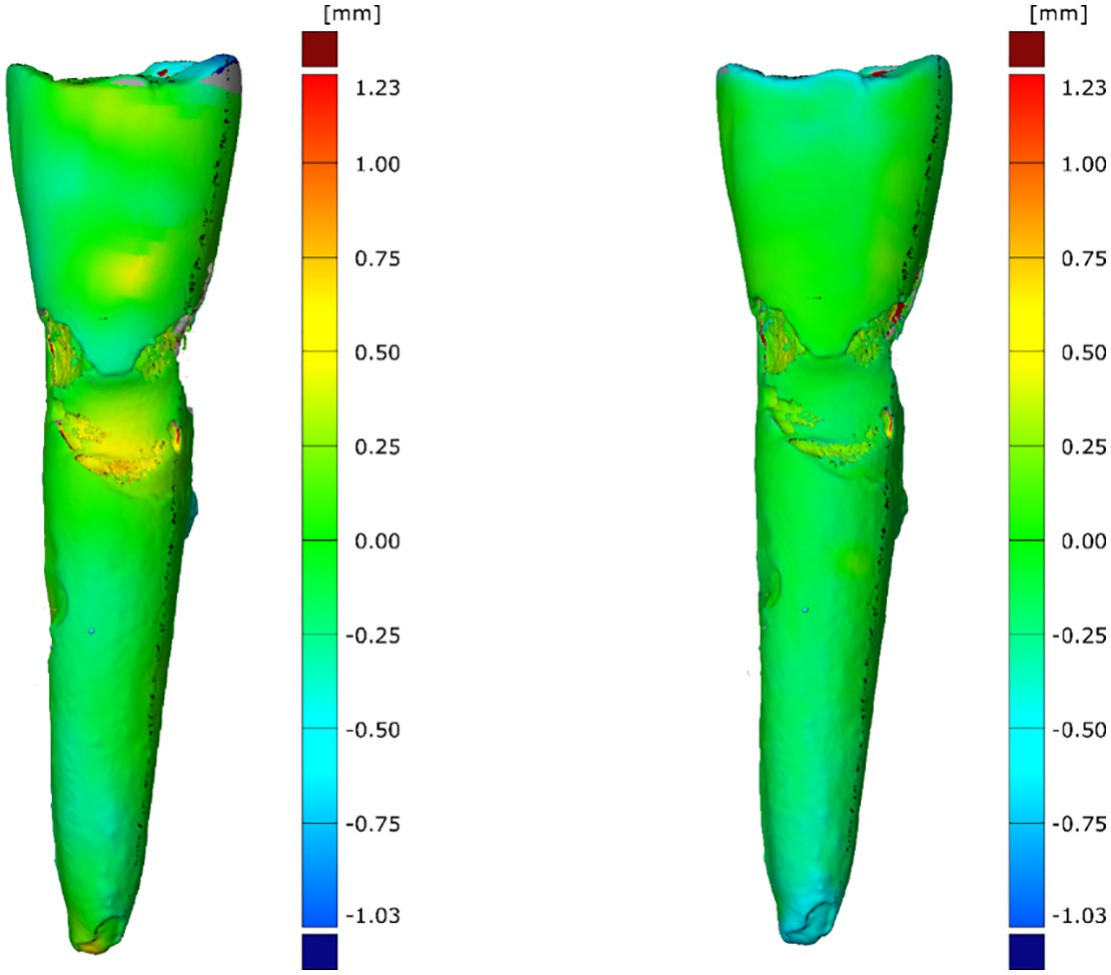
Surface deviations of the three-dimensional models. **(a)** PAT compared with μ-CT imaging. **(b)** CBCT compared with μ-CT imaging.

**Fig 8:**
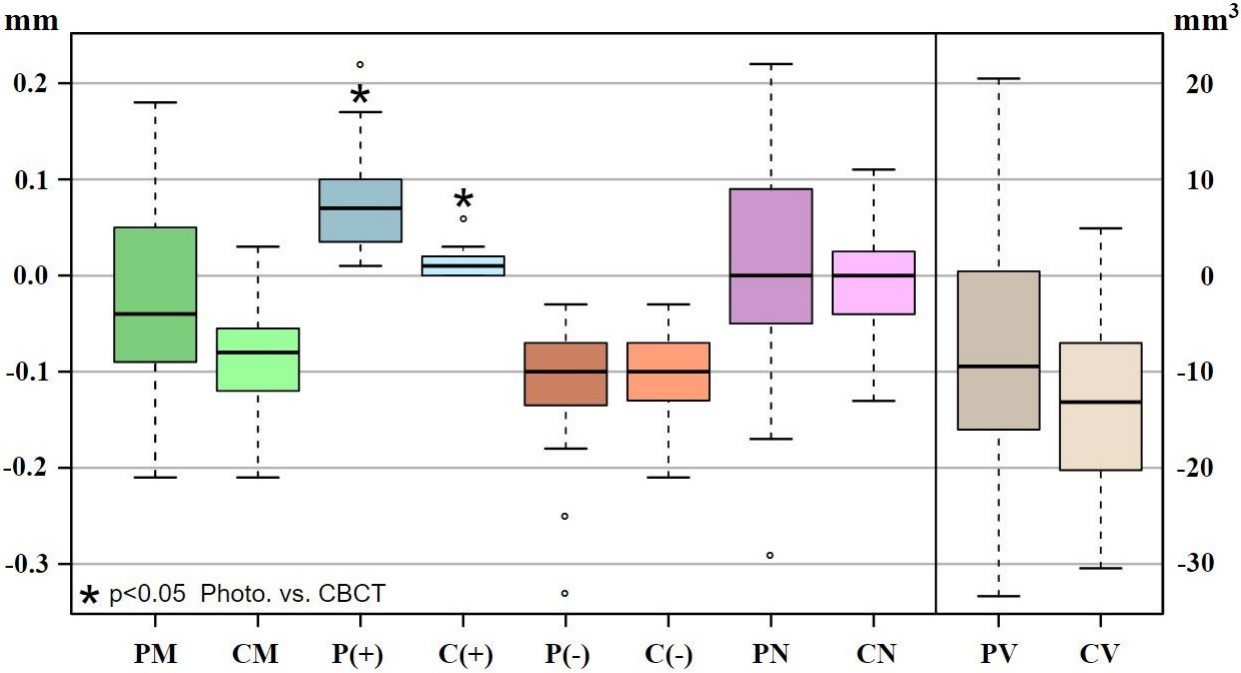
Box-and-whisker plot of results of matching process, with list of abbreviations. Group 1: PAT vs. μ-CT (white); group 2: CBCT vs. μ-CT (blue). Surface and volume deviations for PAT compared with μ-CT, and for CBCT compared with μ-CT. Mandibular incisors were scanned using μ-CT, CBCT, and PAT. Individual three-dimensional models were created. μ-CT images were defined as the reference standard. CBCT and PAT images were compared with μ-CT images. The root surface and volume deviations between PAT and μ-CT, and between CBCT and μ-CT are displayed. * = significant difference between PAT and CBCT. The results for the comparison of PAT with μ-CT had a slightly larger range than the results for the comparison of CBCT with μ-CT. **PM**: **Total average of the deviation of the RSA (PAT compared with μ-CT)**, **CM**: Total average of the deviation of the RSA (CBCT compared with μ-CT), **P (+)**: Positive deviation of the RSA (PAT compared with μ-CT), **C (+)**: Positive deviation of the RSA (CBCT compared with μ-CT), **P (−)**: Negative deviation of the RSA (PAT compared with μ-CT), **C (−)**: Negative deviation of the RSA (CBCT compared with μ-CT), **PN**: Normalized total average of the deviation of the RSA (PAT compared with μ-CT), **CN**: Normalized total average of the deviation of the RSA (CBCT compared with μ-CT), **PV**: Volume deviation of the RSA (PAT compared with μ-CT), **CV**: Volume deviation of the RSA (CBCT compared with μ-CT), **SD**: Standard deviation.

The results of the matching process are displayed in Fig 7 and as a box-and-whisker plot in Fig 8. The absolute values of the lower and the upper quartiles of the surface deviations were all < ±0.15 mm, and the median value of the surface deviations was < ±0.1 mm. The absolute values of the lower and upper quartiles of the volume deviations were all > −20 mm^3^, and the median value of the volume deviations was < −13 mm^3^.

In general, the PAT images had a slightly smaller total surface than the μ-CT images. However, the maximum deviation of the PAT images into the positive territory was 0.22 mm, and the maximum deviation into the negative territory was −0.33 mm. The difference between the PAT–μ-CT group and CBCT–μ-CT group regarding the total average of the root surface area was not significant (p>0.06).

## 4. Discussion

Three-dimensional images are important for diagnosis and treatment planning, and therefore also have a substantial impact on the success rate of medical treatment. As a result, high-resolution imaging and three-dimensional reconstruction of teeth in dentistry have become extremely important. Although non-ionizing imaging techniques are available, X-ray imaging is still used as standard in dentistry.

In the present study, no significant differences were found between the PAT–μ-CT group and CBCT–μ-CT group regarding the total average of the RSA and total volume. Both null hypotheses must therefore be accepted. Nonetheless, the results for the comparison PAT–μ-CT had a larger range than the results for the comparison CBCT–μ-CT (Fig 7). This might be because the ultrasound detection array of the MSOT inVision has an angular convergence of only 270 degrees, whereas CBCT has 360-degree angular coverage. Furthermore, because the teeth were embedded in water and agarose for PAT imaging, it was not possible to reliably prevent them from moving during scanning, which might have led to movement artifacts in some cases. The penetration depth of X-rays is superior to that of near-infrared light, resulting in lower-quality images when PAT is used. This fact is of special interest regarding teeth that have, for example, a thicker layer of root cementum, because the light will not be able to penetrate these teeth as easily as teeth with a thin layer of root cementum.

Unlike US and OCT (Optical Coherence Tomography), PAT utilizes both light and sound to generate an image [14]. If only light is used, the penetration depth is quite limited, because of scattering in the tissue [22]. If only ultrasound is used, a better penetration depth can be achieved, because the scattering effect is reduced. However, the application of US results in poor contrast^22^. The detour from light to sound that occurs in PAT achieves both a higher spatial resolution and a greater penetration depth than OCT or US [14]. However, the spatial resolution of PAT is still limited, because it is affected by various other factors: It is compromised by acoustic inhomogeneity, insufficient data (due to a limited view angle), and a limited bandwidth of the detection system as a result of the size of the detector aperture [23].

Nonetheless, the spatial resolution of PAT is already sufficient to achieve reliable information about early-stage caries, as was shown by a 2016 in-vitro study by Cheng et al. [24]. This finding was supported by Koyama et al., who showed in 2018 that it might be possible to use PAT to detect caries. In that in-vitro study, caries could be detected by using frequency components of 0.5–1.2 MHz on specimens with simulated caries. In caries-free specimens, however, this frequency was not adequate [25].

Because PAT measurements are associated with an increase in temperature in the illuminated tissue, care must be taken to ensure that the pulp of the examined tooth remains undamaged. In a 2006 in-vitro study, Li and Dewhurst showed that the temperature of a postmortem tooth does not increase by more than 5ºC during PAT measurement. This is important information, because Li and Dewhurst found that the pulp starts to necrotize if it is heated by more than 5ºC. Furthermore, Li and Dewhurst demonstrated that it is possible to assess the distribution of caries in postmortem dental samples by using PAT [26].

In their 2006 in-vitro study, El-Sharkawy et. al. used a Nd:YAG laser with a wavelength of 1064 nm to examine the optical and physical properties of teeth. They showed that it is possible to distinguish between an intact and a decayed tooth by calculating the longitudinal sound speed, optical penetration depth, and Grueneisen coefficient of the tooth [27].

Regarding the use of photoacoustics in daily clinical practice in dentistry, several studies have already shown that photoacoustic imaging has potential. In 2018, Moore et al. successfully depicted the gum, gingival margin, and gingival thickness of teeth 7–10 and 22–27 by using PAT in combination with a contrast medium (cuttlefish ink). Furthermore, the study showed that PAT provides a more reliable measurement of periodontal pocket depths than a dental hygienist using a periodontal probe does [28]. In a 2021 study, Mozaffarzadeh et al. [29] used a dual-modality photoacoustic-ultrasound (PA-US) imaging system to resolve periodontal anatomy and periodontal pocket depths in humans. In a second step, the authors managed to reduce motion artifacts and generate artifact-free 2D cross-sections by means of an image-registration technique. In 2022, Lei Fu et al. could show with their study that it is not even possible to display periodontal pockets of anterior teeth, but also of posterior teeth [30]. However none of these studies used PAT to analyze hard dental tissue.

Imaging of hard tissue such as the brain using PAT remains challenging, because hard tissue like the skull and brain tissue of larger mammals such as monkeys generate strong optical scattering. This scattering severely limits optical fluency [31].

Nonetheless, photoacoustic imaging still presents several difficulties when it comes to in-vivo imaging of teeth.^30^ As shown in our study, dense structures such as enamel hinder the optical path. Consequently, not only is it difficult for this path to overcome dental materials such as amalgam, composite, metal pins, brackets, and dental crowns, but also the jawbone surrounding the teeth. To avoid severe image distortion, it is preferable to avoid all dental materials that can be excluded, such as amalgam, composite, metal pins, brackets, and dental crowns, in both the tooth of interest and its antagonists and neighbors. However, it is conceivable that the reconstruction algorithm could be further improved, which could enable imaging of dense structures.

To make PAT a viable option in clinical practice, two problems need to be overcome: (1) the visualization of dense structures (as discussed above) and (2) the length of acquisition time, which needs to be reduced to enhance patient comfort during imaging. The results of this study indicate that the acquisition time can be reduced from more than nine minutes to less than one minute, by using only one suitable single wavelength (680 nm) instead of various wavelengths.

Furthermore, a suitable imaging device is needed. Photoacoustic imaging systems for clinical research, e.g., the MSOT Acuity, are conceivable options here. Because it has a frequency of 25 Hz, the MSOT Acuity has the potential to record images twice as fast as the MSOTin Vision 256, which would permit real-time imaging. To achieve an imaging depth of up to 3 cm, the imaging device could be placed on the skin. Here, the distance between the scanned object, laser, and detector should be kept as small as possible and, if possible, the three components should be connected by a suitable medium, e.g., water, which could be swallowed afterwards by the patient.

Several limitation have to be considered when interpreting the results of this in-vitro study.

First, the teeth were embedded in artificial fluids, not representing the environment in the oral cavity. Thus, imaging teeth in-vivo might be more complex and the results of an in-vitro study have to be interpreted with care.

Second, PAT image acquisition in-vitro is much easier, that the acquisition in-vivo due to anatomical limitations.

Finally, another limitation of the present study should be critically discussed: a 270 degree array was used to acquire PAT images of the entire tooth in-vitro. However, this setup cannot be transferred to all clinical conditions. In the lower incisors, a 270 degree array might be used clinically, whereas in other teeth (premolars, molars), this approach might be impossible due to the anatomical situation.

However, in-vitro studies are helpful to assess the validity and reliability of imaging modalities when compared to a goldstandard [10], although there are inevitable limitations of in-vitro studies in general.

## 5. Conclusion

Images, which were acquired using PAT at 680nm showed the best contrast ration, enabling the identification of dentin, cementum and the dental pulp. No significant differences were found between the PAT–μ-CT group and CBCT–μ-CT group regarding the total average of the RSA and the total volume. Thus, three-dimensional reconstructions based on in-vitro PAT are already of acceptable reconstruction quality.
